# Investigation of Mechanochromic and Solvatochromic Luminescence of Cyclometalated Heteroleptic Platinum(II) Complexes with Benzoylthiourea Derivatives

**DOI:** 10.3390/molecules30112415

**Published:** 2025-05-31

**Authors:** Monica Iliş, Marilena Ferbinteanu, Cristina Tablet, Viorel Cîrcu

**Affiliations:** 1 Department of Inorganic and Organic Chemistry, Biochemistry and Catalysis, University of Bucharest, 4-12 Regina Elisabeta Bld., Sector 5, 030018 Bucharest, Romania; monica.ilis@chimie.unibuc.ro (M.I.); marilena.cimpoesu@g.unibuc.ro (M.F.); 2 Faculty of Pharmacy, Titu Maiorescu University, Gh. Sincai Bd. 16, 040317 Bucharest, Romania; cristina.tablet@prof.utm.ro

**Keywords:** platinum(II) complexes, cyclometalation, luminescence, benzoylthiourea, mechanochromism

## Abstract

Two novel cyclometalated platinum(II) complexes based on 2-phenylpyridine (ppy) and 2,4-difluorophenylpyridine (dfppy) ligands in combination with a benzoylthiourea (4-(decyloxy)-N-((4-(decyloxy)phenyl)carbamothioyl)benzamide, BTU) functionalized with decyloxy alkyl chains as auxiliary ligands were synthesized and characterized for their mechanochromic and photophysical properties. Structural characterization was achieved through IR and NMR spectroscopy, single-crystal X-ray diffraction, and TD-DFT calculations. Both complexes exhibit significant photoluminescence with quantum yields up to 28.3% in a 1% PMMA film. The transitions in solution-phase spectra were assigned to mixed metal-to-ligand (MLCT) and intraligand (ILCT) charge–transfer characteristics. Temperature-dependent studies and thermal analyses confirm reversible phase transitions without mesomorphic behavior despite the presence of the two long alkyl chains. Both complexes displayed reversible mechanochromic and solvatochromic luminescence, with a change in emission color from green to red-orange emissions upon grinding and solvent treatment or heating at 80 °C.

## 1. Introduction

Mechanochromism is the reversible or irreversible change in color or luminescence of a material resulting from mechanical stimulation—that is, from grinding, pressing, or shearing [1,2]. Platinum(II) complexes have attracted a lot of interest in the field of coordination chemistry mostly because of their square planar geometry, which allows strong intermolecular interactions such as π–π stacking and Pt···Pt metallophilic contacts. These highly sensitive to external mechanical forces interactions cause changes in the solid-state packing and hence the photophysical characteristics of the complexes [3]. Often the mechanochromic behavior in Pt(II) complexes is ascribed to changes in their aggregation state. Mechanical grinding, for example, can disturb the crystalline order and turn it into an amorphous condition, changing the Pt···Pt distances and the π–π interactions, and consequently, significant changes in emission wavelengths and intensities can follow from this modification [4]. The design of Pt(II) complexes with particular ligands to improve mechanochromic responses has been an ongoing topic of research in light of their possible applications in chemical sensors [5,6,7], security inks [8,9], and smart display technologies [10,11] due to their adjustable mechanochromic characteristics. On account of their high emission efficiency, room temperature phosphorescence, and square planar shape, cyclometalated platinum(II) complexes have emerged as one of the most appealing research fields among emissive metal complexes [12]. Furthermore, it is well known that by exposing these phosphorescent cyclometalated Pt(II) complexes to specific external environments, it is possible to adjust their solid-state properties in a targeted manner. The color and luminescence of Pt(II) compounds may change noticeably when exposed to mechanical stress, heat, and organic solvents due to the development of efficient metallophilic, π···π, or C-H···π interactions, which produce redshifted emissions [13,14]. Research on platinum(II)-phenylpyridine (Pt-ppy) complexes proves the crucial importance of cyclometalation as an effective tool to improve the photophysical properties of related platinum complexes. The strong ligand field of cyclometalating carbon raises the energy of the non-emitting metal-centered (d-d) excited states above the triplet energy of the cyclometalated ligand, leading to a great increase in quantum efficiencies [15]. This study proposes the investigation of the mechanochromic properties of a new family of heteroleptic Pt-ppy complexes based on benzoylthiourea auxiliary ligands (BTU). In coordination chemistry, benzoylthiourea or, more generally, acylthiourea ligands are a class of structurally varied and functionally rich ligands distinguished by their capacity to coordinate transition metals through both nitrogen and sulfur donor atoms. Due to thione sulfur, the thiourea moiety imparts a soft donor character; the adjacent amide nitrogen helps chelate, enabling bidentate (S, N) coordination [16]. The benzoyl group introduces substituents to enable the fine-tuning of solubility, electronic properties, and biological or photophysical activity, as well as additional electronic and steric tunability, improving the ligand’s capacity for metal complexation [17,18].

Due to these properties, benzoylthiourea ligands are extensively used to coordinate a variety of metal centers, including copper, nickel, cobalt, zinc, and platinum, forming complexes with applications in catalysis, bioinorganic chemistry, and materials science. In the design of metal complexes with specific functional properties such as redox activity, biological interaction, or photoluminescence, their great chelating ability combined with structural flexibility and π-conjugation makes them particularly valuable [19].

**Scheme 1:**
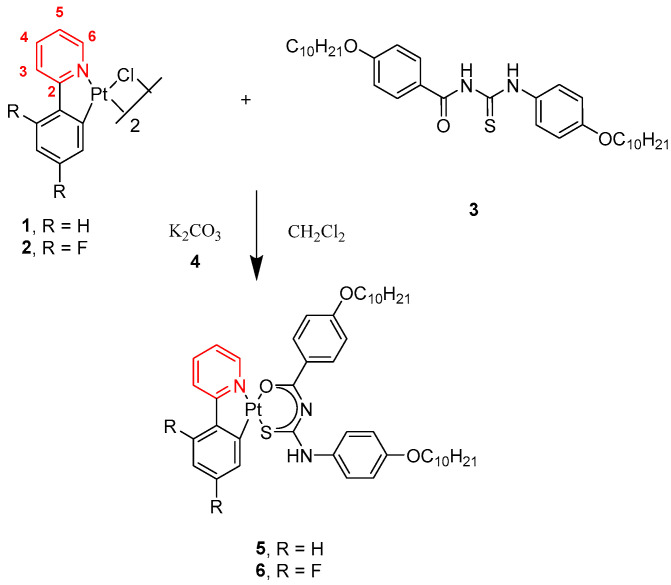
Chemical structure and synthetic pathway of Pt(II) complexes.

In the present work, we investigate the synthesis, crystal structure, stimuli-responsive luminescent behavior, and changes in emission colors induced by two novel cyclometalated Pt-ppy complexes (ppy = 2-phenylpyridine (2-ppy) or 2,4-difluorophenylpyridine (2dfppy)), with a BTU auxiliary ligand, 4-(decyloxy)-N-((4-(decyloxy)phenyl)carbamothioyl)benzamide, similar to those reported by us (Scheme 1) [20,21].

## 2. Results and Discussion

### 2.1. Synthesis and Structure of Pt Complexes

The mononuclear cyclometalated Pt(II) complexes were synthesized by reacting the platinum precursors **1** and **2** in dichloromethane with the BTU ligand (**3**) [20,21,22] in the presence of K_2_CO_3_. After purification on silica, the previously unknown mononuclear compounds **5** and **6** were produced in high yields (Scheme 1). These complexes are yellow microcrystalline solids that are stable in ambient conditions, and no changes in their color, emissions, or texture were observed over a period of five years.

Elemental analysis studies, IR spectroscopy, ^1^H and ^13^C NMR spectroscopy, and single-crystal X-ray diffraction all validated their structure. For example, in the ^1^H-NMR spectra of the two Pt complexes, the CH-6 proton of pyridine ring of the chelating 2-ppy or 2-dfppy ligands appeared as a broad doublet in the range of 9.17–9.25 ppm with the coupling constants ^3^*J*(^195^Pt–^1^H) of 29.2–31.3 Hz, which confirms the orthometalation of the ligands. The target complexes contain a Pt atom with one ortho-metallated 2-ppy derivative and one bidentate BTU ligand, presumably with sulfur atoms in a trans position with respect to the nitrogen of the ppy fragment, as revealed by ^1^H-NMR spectroscopy. There is a direct analogy with the chemical structure of the previously isolated Pt-ppy complexes with simple BTU ligands confirmed by single-crystal X-ray diffraction studies [23,24,25]. Given that the ^1^H-NMR spectra show one set of signals with similar values of chemical shifts in aromatic protons, it was fairly assumed that these complexes have the same arrangement of coordinating atoms around the Pt center (Figure S1, Supplementary Materials) [26].

**Figure 1:**
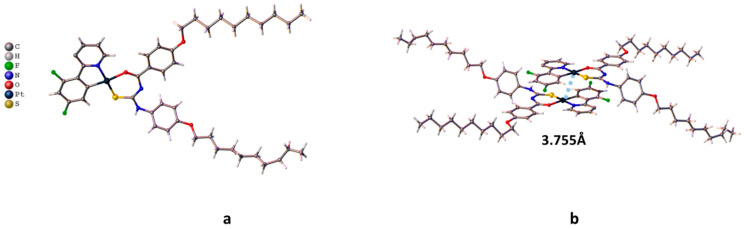
Molecular structure of compound **6** (**a**) and its packing of dimers in the crystal lattice (**b**).

To confirm the coordination of BTU ligands to platinum, several attempts to obtain single crystals for X-ray analysis were made. Suitable single crystals of complex **6** were successfully grown from a mixture of acetone and methanol at −25 °C. The molecular structure for **6** is shown in Figure 1.

**Figure 2:**
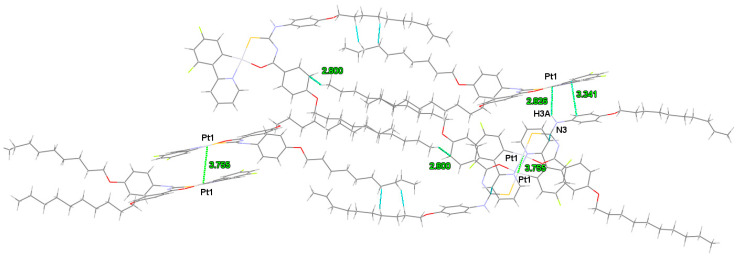
Packing diagram to show the Pt^…^Pt, C-H^…^π contacts and π-π intermolecular interactions for **6**.

Crystallographic information is presented in Table 1, whereas the selected bond lengths and angles for **6** are collected in Table 2. Complex **6** crystallized in the monoclinic crystal system, space group *P*2_1_/c, with a square planar arrangement of the platinum atom, which was surrounded by one aromatic carbon atom (a metallated phenyl ring of 2-dfppy), one nitrogen atom (a pyridine ring of 2-dfppy), one oxygen atom of the carbonyl group, and one sulfur atom of the thiocarbonyl group (thiourea) in a trans position versus the nitrogen atom. The bond lengths of Pt-C (1.964(11)Å), Pt-N(2.009(12)Å), Pt-O (2.046(11)Å), and Pt-S (2.243(5)Å) are similar to those found for cyclometalated Pt-ppy complexes [27] and Pt complexes with benzoylthiourea ligands [23]. While the aromatic ring of the carbonyl moiety is almost coplanar with the dfppy ligand, the phenyl ring linked to the thiocarbonyl group is twisted about 119° in this respect and almost parallel to the dfppy unit of a neighboring molecule to produce π-π interactions with a dppy/Ph separation of 3.341 Å (Figure 2). The orientation of the two arms of the BTU ligand points toward a closed geometry in a solid state.

The crystal packing of complex **6** reveals an antiparallel stacking pattern along the vertical axis of each molecule, resulting in a dimerlike structure. The shortest intermolecular Pt^…^Pt distance found within the unit cell was 3.755 Å, while the next shortest Pt^…^Pt distance was measured to be 7.184 Å. The assembly of a 1D infinite chainlike packing arrangement, which has been observed for many Pt(II) complexes, was ruled out by such substantial intermetallic distances [28]. There were fewer significant Pt-Pt interactions in **6** since the intermolecular Pt^...^Pt distances were greater than double the van der Waals radius of Pt (3.50 Å) [29]. The significant close contacts N-H^…^Pt, C-H^…^π (C-H_Alk_^…^Ph(BTU)), and π-π (dfppy^...^Ph(BTU)) of 2.826 Å, 2.800 Å, and 3.341 Å, respectively, were found by close examination of the crystal packing of **6** (Figure 2).

### 2.2. Thermal Characterization of Pt Complexes

**Figure 3:**
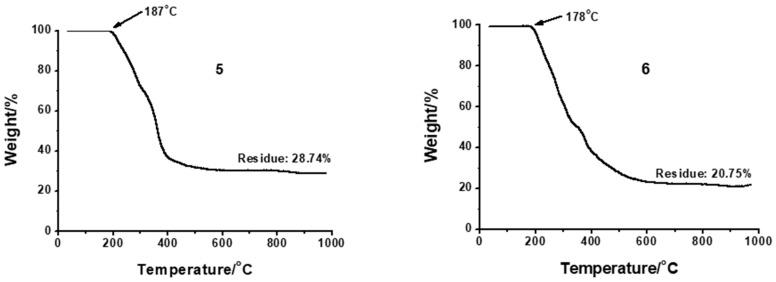
TG curves for complexes **5** and **6**.

**Figure 4:**
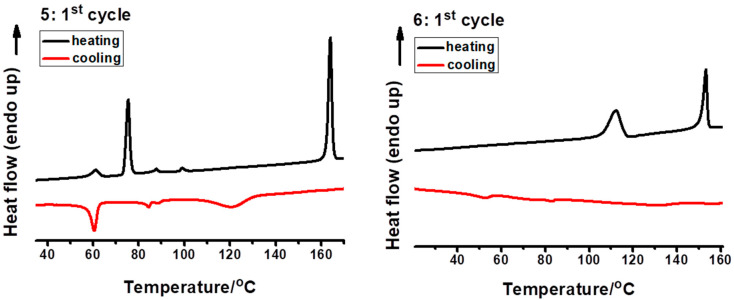
DSC thermograms for **5** and **6**.

The two Pt complexes were subjected to a TGA (thermogravimetric analysis) study, which reveals that their decomposition starts around 200 °C, well above their melting temperatures (Figure 3). Further, the thermal behavior of the Pt complexes was analyzed by DSC and polarized optical microscopy (Figure 4). The corresponding thermal data (phase transition temperatures and enthalpies) are summarized in Table 3.

The Pt compounds exhibit multiple transitions between distinct crystalline phases during heating and cooling operations (Figure 4). Owing to the presence of a long alkyl chain on the BTU ligand, the complexes were investigated for the presence of mesophases.

**Figure 5:**
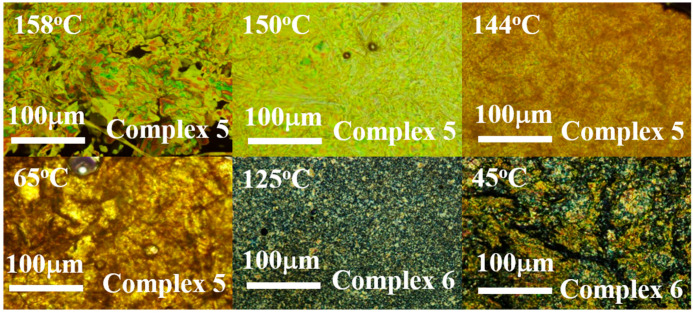
POM pictures for **5** and **6** taken on cooling from the isotropic state at different temperatures.

Thus, additional POM observations did not evidence any liquid crystalline textures for complexes **5** and **6** (Figure 5), confirming only the presence of various crystalline phases. Although the presence of long alkyl chains is a common design strategy for inducing mesomorphism, the strong intermolecular interactions, lack of sufficient phase segregation, and molecular shape likely prevent the formation of liquid crystalline phases for the two Pt complexes.

### 2.3. Photophysical Characterization

The photophysical characterization of the platinum(II) complexes **5** and **6** was carried out at room temperature in aerated CH_2_Cl_2_, PMMA films and in the solid state. All data are summarized in Table 4. The Pt complexes show high solubility in common organic solvents, including CH_2_Cl_2,_ which allowed their characterization in solution.

**Figure 6:**
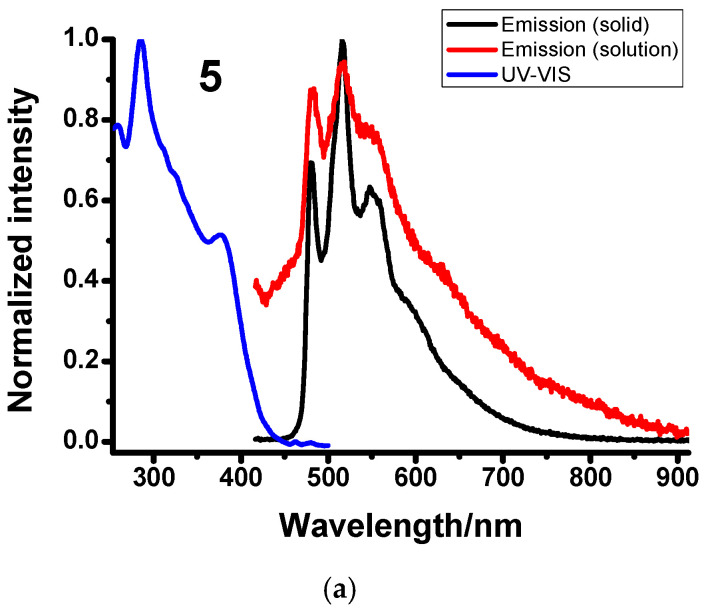

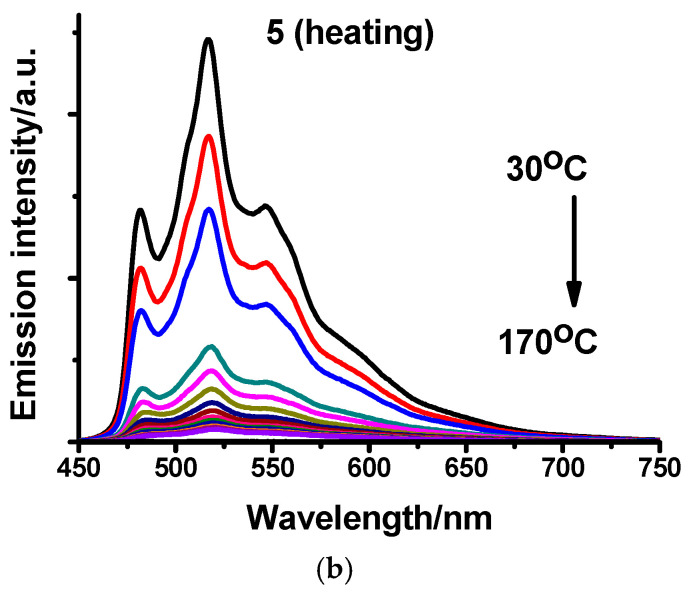
UV-VIS and emission spectra in a solid state and solution in dichloromethane for **5** (**a**) and temperature-dependent emissions for **5** (**b**).

**Figure 7:**
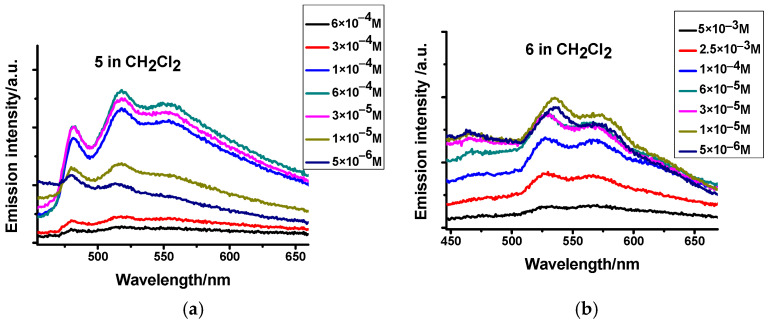
The emission spectra of **5** (**a**) and **6** (**b**) in dichloromethane at various concentrations.

The UV-VIS and emission spectra in the solid state and solution for complexes **5** and **6** are presented in Figure 6 and Figure 7. The Pt complexes exhibit distinct absorption spectra with highly intense transitions (ε > 13 × 10^3^ M^−1^·cm^−1^) that are attributed to intraligand charge–transfer transitions (^1^ILCT) throughout the wavelength range of 250–350 nm. Transitions into states with a substantial metal-to-ligand charge–transfer (^1^MLCT) character mixed with ^1^ILCT contributions are responsible for the low-energy absorption features in the 325–400 nm region (Table 5 and Figure S6, Supplementary Materials). These characteristics have been correlated to lower molar absorption coefficients. To better understand the characteristics of absorption and emission, density functional theory (DFT) modeling of the two Pt complexes was performed at a PBE0/MWB60/6-31G** level of theory.

**Figure 8:**
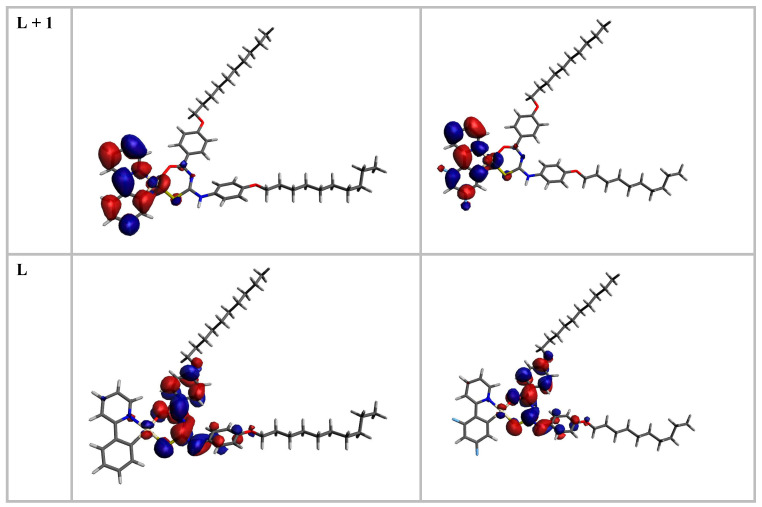

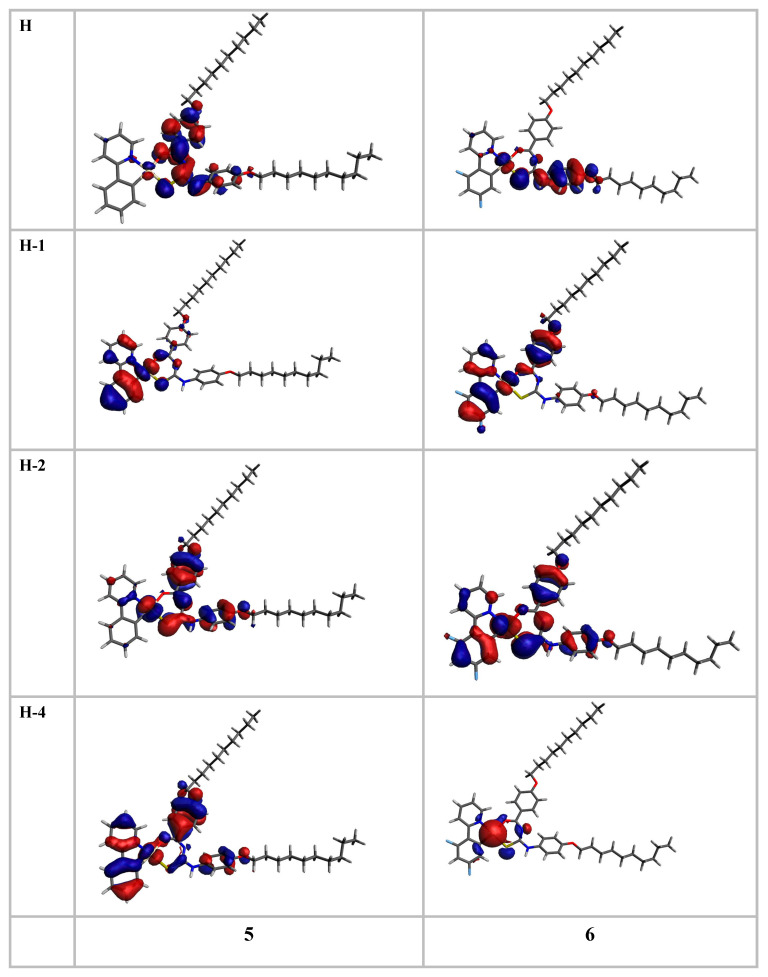
Shapes of selected molecular orbitals in the S_0_ state (isodensity value of 0.02 e/B^3^) for all studied complexes in CH_2_Cl_2_. **H** stands for the highest occupied molecular orbital, and **L** stands for the lowest unoccupied molecular orbital.

According to DFT and time-dependent TD-DFT, the low-energy absorption (S_0_ → S_1_) of the two Pt complexes is predominantly originating from the HOMO → LUMO transition (Figure 8 and Table 5). The HOMO of each complex exhibits notable contributions from Pt, 15.48% and 11.70% for **5** and **6**, respectively, as well as from the BTU ligand, specifically the PhNHC=S fragment (~70%) and the OC_10_H_21_ group of the PhNHC=S arm (~10%) (see Table 6).

The emission bands of **5** and **6** have a distinct vibrational structure, with a spacing of approximately 1350 cm^−1^, which corresponds well to the frequency of vibrations in aromatic systems. Platinum complexes often exhibit aggregate/excimer emission as a result of intermolecular Pt⋯Pt interactions and π-π or C-H⋯π interactions [29,30,31]. Further, this study focused on investigating the potential intermolecular interaction by examining the concentration-dependent luminescence of Pt compounds. The emission maxima of complexes **5** and **6** display minimal variation with increasing solution concentration, while the emission intensity diminishes at higher concentrations due to aggregation-caused quenching. The low quantum yield values observed in the solution can be attributed to the measurements conducted in aerated solvents.

**Figure 9:**
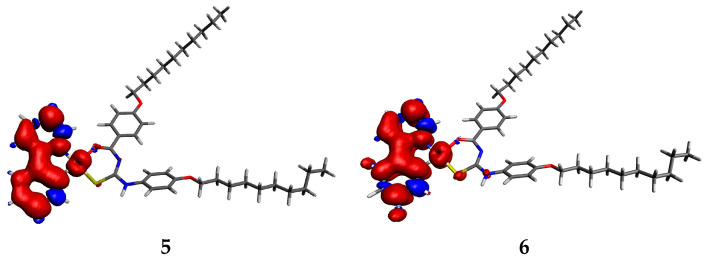
Spin density plots (isodensity value of 0.001 e/B^3^) for T_1_ optimized states in CH_2_Cl_2_ for Pt complexes.

Hence, the presence of the electron-acceptor groups on the dfppy ligand produced the blue shift in the emission maximum from 480 nm for **5** to 467 nm for **6**. The calculated spin-density distribution in the optimized T_1_ state in CH_2_Cl_2_ (Pt of 0.2062 for **5** and 0.1722 for **6**, Table 7 and Figure 9), as well as the HOMO-1 and LUMO+1 orbitals (Table 6 and Figure 8), are located on the 2-dfppy and Pt and, therefore, evidence a mixture of ^3^ILCT/^3^MLCT with a small contribution from a BTU ligand (Table 8. According to this assignment, the variation in the cyclometalated ligand has a strong impact on the emission of the two complexes in the CH_2_Cl_2_ solution, as reported for other ppy and dfppy cyclometalated Pt complexes [27,31,32,33,34]. The solid-state emission is dominated by a distinct band with a vibronic pattern similar to that observed in the solution and no significant shift or the emission maxima.

Further, temperature-dependent studies were conducted to analyze the luminescence and self-assembly behavior of the Pt complexes. Complexes **5** and **6** display intense green emissions on both repeated heating and cooling cycles, with their emissions quenched during heating to isotropic states and restored when cooling to crystalline phases (Figure 6b).

We then investigated the impact of additional external stimuli, in particular mechanical action, based on these findings, which occur when the emission color of the Pt complexes varies according to their aggregation state (crystalline or amorphous) [1,2,35]. The emission colors of mechanochromic luminescent materials are altered when the appropriate pressure or other mechanical force is applied to the material. Examples of such forces include grinding, crushing, rubbing, or extrusion. It is possible for these substances to return to their initial condition with the application of an additional external alteration stimulus. The intention of the investigation was to determine whether the samples might undergo a transformation from their amorphous to crystalline state using organic vapor adsorption or heating while simultaneously changing their red-orange luminescence to green emissions. Different substituents that present various steric hindrances cause molecules to arrange themselves in different ways, which then leads to different mechano-induced changes in color and luminescence. The same effect would also happen with compounds that have different electron-withdrawing or electron-donating substituents.

**Figure 10:**
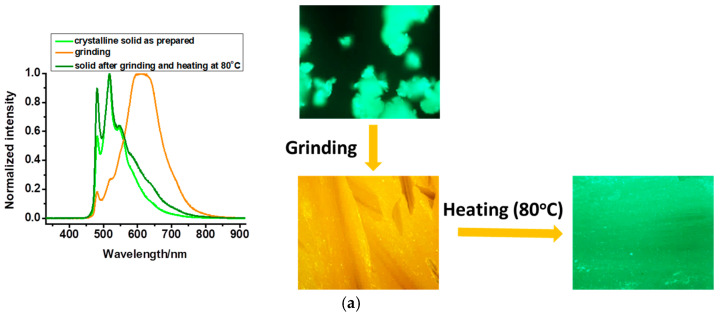

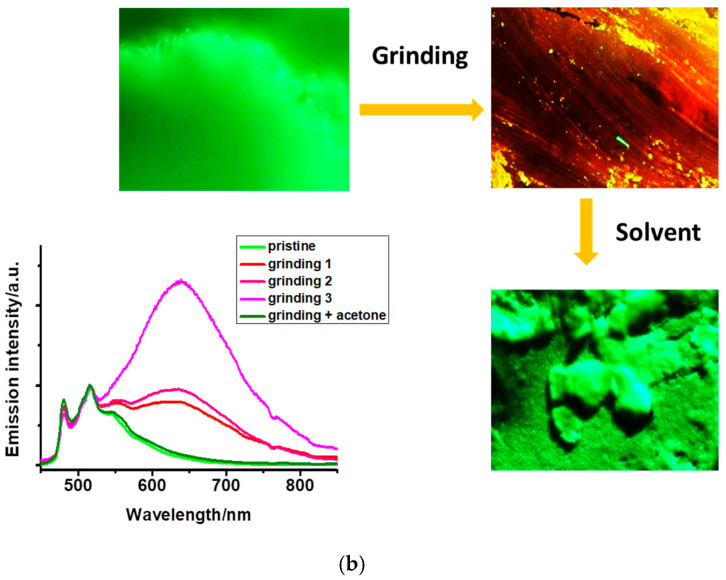
Mechano- and solvatochromism properties of **5** (**a**) and **6** (**b**).

The two Pt complexes show similar mechano- and solvatochromism properties (Figure 10). By grinding or scratching the samples with a spatula on the glass substrate, the initial green emissions of the crystalline products can be quickly changed to orange emissions. After treating the ground samples with different solvents (acetone or ethanol) or by heating around 80 °C, the emission spectra returned to their original state, as shown in Figure 10. The repetition of this cycle reveals that the mechanochromic change in luminescence is reversible.

## 3. Materials and Methods

### 3.1. Materials and Characterization

All chemicals were purchased from Merck and used without further purification. CH_2_Cl_2_ was purified via distillation under nitrogen after stirring for 2 h in the presence of P_2_O_5_. Column chromatography was performed on a 200−300 mesh silica gel and with dichloromethane/pentane (4/1 *v*/*v*) as an eluting solvent to purify the synthesized Pt(II) complexes. The BTU auxiliary ligand (**3**) was prepared following the procedure reported earlier by us [20,21]. Nuclear magnetic resonance (^1^H NMR and ^13^C NMR) spectra were recorded in CDCl_3_ on a 300 or 400 MHz spectrometer (Bruker BioSpin NMR, Rheinstetten, Germany) using residual solvent peaks as reference. Elemental analysis was performed on an element analyzer (EURO EA 3300, Eurovector, Pavia, Italy). A Bruker Tensor V-37 spectrophotometer (Bruker Optics Inc., Billerica, MA, USA) was employed for recording the IR spectra of all compounds in the range of 4000–400 cm^−1^ in KBr discs. UV-VIS spectra were recorded using a Jasco V-630 spectrophotometer (JASCO Corp., Tokyo, Japan) in dichlorometane solutions with a 10^−4^ M concentration. Dichloromethane is a common solvent used for UV-VIS and emission measurements, and it ensured the best dissolution of the Pt samples. Luminescence quantum yields in non-degassed dichloromethane solutions were determined with OceanOptics QE65PRO spectrometer (Ocean Optics Inc., Orlando, FL, USA) at an excitation wavelength of 365 nm and an LED source (LLS-LED, OceanOptics, Ocean Optics Inc., Orlando, FL, USA). The optical density of all solutions was about 0.1 at the excitation wavelength (365 nm). [Ru(bpy)_3_]Cl_2_ in air-equilibrated aqueous solution (Φ = 0.02827) was used as the standard; the estimated uncertainty in Φ was 15% or better. The emission spectra, absolute quantum yields (QYs), and lifetimes in a solid state were measured on an FS5 spectrofluorometer (Edinburgh Instruments). The variable temperature photoluminescence spectra were recorded for the samples deposited as films on a glass slide, with the OceanOptics QE65PRO spectrometer (Ocean Optics Inc., Orlando, FL, USA) attached to the polarizing optical microscope and using a Nikon Intensilight excitation source and UV-2A filter (excitation range of 330–380 nm). Doped PMMA films for emission measurements were prepared by dissolving 99 mg of the polymer and 1 mg of each Pt compound in 2 mL of dichloromethane with a typical concentration of the complex in PMMA of 1%. The films were obtained by the drop-casting method using glass substrates and drying at room temperature for 24 h before recording the emission properties.

The thermal stability of Pt(II) complexes was measured in an inert atmosphere (N_2_, 20 mL/min) on a thermogravimetric analyzer (STA 6000, Simultaneous Thermal Analyzer, Perkin Elmer, Boston, MA, USA) at a heating rate of 10 °C/min from 30 to 950 °C, using alumina crucibles. The phase transition temperatures and corresponding enthalpies were measured in a nitrogen atmosphere using differential scanning calorimetry (DSC Diamond, Perkin Elmer, Boston, MA, USA) with a scanning rate of 10 °C/min in the 0 to 200 °C temperature range. The samples were encapsulated in aluminum pans. Polarized optical microscopy (POM) images were captured using a microscope (50iPol, Nikon Instruments, Melville, NY, USA) and a hot stage (THMS 600, Linkam Scientific Instruments Ltd., Tadworth, UK) connected to a temperature controller (TMS 94). Untreated glass slides were used for POM observations. POM textures were captured when cooling the isotropic liquid samples down to room temperature at a ramp rate of 10 °C/min.

Single-crystal XRD data collection: The crystal data were collected on a Rigaku R-AXIS RAPID II diffractometer (Rigaku, Japan) using graphite monochromated Mo-Kα radiation (λ = 0.71075 Å) and the ω-ϕ scan technique at room temperature. A yellow plate crystal of C_45_H_55_F_2_N_3_O_3_PtS (**6**) with the approximate dimensions of a 0.400 × 0.300 × 0.050 mm single crystal was mounted on a glass fiber. The data were collected with the Crystal Clear program. The structure was solved by direct methods [36], and the non-hydrogen atoms were refined anisotropically using a full-matrix least-squares method based on *F*^2^. Hydrogen atoms were refined using the riding model. The data were corrected for Lorentz and polarization effects. Calculations were performed using the Olex2 1.5 software package [37]. The packing crystal details are presented in Figure 2 and Figure S4 (Supplementary Materials). CCDC 2350718 includes all the supplementary crystallographic data of compound **6**.

Theoretical calculations: Gaussian09 [38] was used to perform DFT and TD-DFT calculations with the hybrid PBE0 functional [39]. The MWB60 and 6-31G** basis sets were considered for the Pt atom and C, H, O, S, F, respectively. Since only the X-ray structure of **6** was available, it was modified and used as the input geometry for all other complexes investigated. Within the PCM model [40], the ground state singlet (S_0_) and the first excited state triplet (T_1_) were optimized in the presence of the dichloromethane solvent and in vacuum. The Δ-SCF method was used for T_1_ optimization. The reliability of the optimized geometries was verified by frequency analysis. In this respect, the optimized geometry corresponds to a minimum on the surface potential curve if it does not lead to an imaginary frequency. The molecular orbitals and spin density shapes were visualized with Avogadro software version 1.2.0 [41]. The contributions of the different fragments to the molecular orbitals were calculated using Multiwfn [42].

### 3.2. Mechanochromic and Solvatochromic Tests

The solid samples (<1 mg) were spread on glass plates (1 × 1 cm). The mechanochromic tests were performed by gentle hand grinding with a spatula until the luminescence color was homogeneous. For solvatochromic tests, one drop of the chosen solvent (ethanol or acetone) was placed on the ground samples, and the luminescence spectra and photographs were recorded after 30 s. Luminescence photographs of samples were taken by a Nikon 50iPol microscope equipped with a DS-1 color camera. The light source of the microscope was a Nikon Intensilight C-HGFI and a filter UV-2A, which passed light in the range of 330−380 nm.

### 3.3. Chemical Synthesis of the Pt(II) Complexes ***5*** and ***6***

A mixture of the corresponding cyclometalating ligand 2-ppy (400 mg) or 2-dfppy (497 mg), 2.6 mmol, and K_2_[PtCl_4_] (457 mg, 1.1 mmol) in a mixture of acetone (15 mL) and water (5 mL) was heated under reflux for 24 h. After cooling down to room temperature, the precipitates of platinum precursors **1** and **2** were collected by filtration and washed with water and hexane [43]. The yellow solids were used in the next step without any further purification. The *N*-benzoylthiourea derivative (**3**) (30 mmol) was added to a solution of **1** or **2** (12 mmol) and K_2_CO_3_ in dichloromethane (15 mL), and the mixture was stirred at room temperature for 48 h. The solvent was removed via rotary evaporation. The residue was purified by chromatography on silica using dichloromethane as eluent and recrystallized from a mixture of acetone/methanol (1/1) at −25 °C.

The yields, elemental analysis results, and the ^1^H and ^13^C-NMR and IR data are presented below:

*Characterization of* **5**. Yield 82%. Anal. Calcd for C_45_H_59_N_3_O_3_PtS: C, 58.93; N, 4.58; H, 6.48. Found: C, 59.07; N, 4.30; H, 6.30. ^1^H NMR (300 MHz, ppm, CDCl_3_) δ 9.24 (d, *J* = 5.2 Hz, *^3^J*_195Pt-1H_ = 31.3 Hz, 1H), 8.14–8.0 (m, br, 3H), 7.90 (t, *J* = 7.7 Hz, 1H), 7.75 (d, *J* = 8.1 Hz, 1H), 7.58–7.39 (m, 3H), 7.35 (t, *J* = 6.0 Hz, 1H), 7.07 (s, br, 2H), 6.91 (m, br, 4H), 4.00 (m, 4H), 1.81 (m, 4H), 1.52–1.20 (m, 28H), 0.89 (t, *J* = 6.5 Hz, 6H). ^13^C NMR (125 MHz, CDCl_3_) δ 165.39, 162.23, 144.74, 144.65, 138.20, 132.72, 131.62, 130.02, 129.77, 124.66, 123.68, 123.54, 121.87, 118.71, 114.54, 114.07, 68.31, 68.19, 31.89, 31.88, 29.68, 29.58, 29.56, 29.54, 29.42, 29.37, 29.31, 29.30, 29.16, 26.06, 26.00, 22.67, 14.10. IR (KBr, cm^−1^): 3304, 3045, 2922, 2852, 1605, 1423, 1247, 749.

*Characterization of* **6**. Yield 70%. Anal. Calcd for C_45_H_57_ F_2_N_3_O_3_PtS: C, 56.71; N, 4.41; H, 6.03. Found: C,56.30; N, 3.96; H, 5.83. ^1^H NMR (300 MHz, ppm, CDCl_3_) δ 9.25 (d, br, *J* = 5.1 Hz, *^3^J*_195Pt-1H_ = 29.2Hz, 1H), 8.21–8.0 (m, br, 3H), 7.92 (t, *J* = 8.1 Hz, 1H), 7.43 (d, *J* = 8.9 Hz, 2H), 7.36 (t, *J* = 5.8 Hz, 1H), 6.99–6.86 (m, 5H), 6.55 (m, 1H), 4.00 (m, 4H), 1.83–1.77 (m, 4H), 1.56–1.20 (m, 28H), 0.89 (t, *J* = 6.5 Hz, 6H). IR (KBr, cm^−1^): 3314, 2920, 2851, 1604, 1434, 1249, 988.

## 4. Conclusions

Two new heteroleptic cyclometalated platinum(II) complexes integrating either 2-phenylpyridine (ppy) or 2,4-difluorophenylpyridine (dfppy) as the cyclometalating ligands and a BTU auxiliary ligand were successfully synthesized and characterized in this work. NMR spectroscopy, elemental analysis, and IR data all support the formation of stable, microcrystalline compounds with long-term ambient stability and high purity from the synthetic strategy. Essential for knowledge of their aggregation-driven optical behavior, single-crystal X-ray diffraction of complex **6** confirms the square planar geometry around the platinum center and reveals important π–π and C–H…π interactions in the solid state. Attributed to the effect of the long alkyl chains on the BTU ligand, the thermal analysis confirms the existence of several solid–solid phase transitions lacking mesophases.

The strong impact of the ligand structure on the emission properties of the complexes was shown by photophysical studies in a solid state, PMMA films, and a solution. Consistent with the electron-withdrawing character of the fluorine substituents, the dfppy-containing complex **6** showed blue-shifted emissions relative to the ppy-based complex **5** in solution. Complex **5** has the highest quantum yield of 28.3% in PMMA films.

Most importantly, both complexes show solvatochromism and reversible mechanochromism. Mechanical grinding converted the luminescence from green (a crystalline phase) to orange (an amorphous state), attributed to the formation of aggregates/excimers, and mild heating or organic solvents help to restore the original emission. Under external stimuli, this reversible behavior suggests a clear structure–property relationship motivated by the modulation of molecular packing and intermolecular interactions.

## Figures and Tables

**Table 1:** Crystallographic data for complex **6**.

Identification Code	Cvlcpt
Empirical formula	C_45_H_57_F_2_N_3_O_3_PtS
Formula weight	953.08
Temperature/K	293.00
Crystal system	monoclinic
Space group	P2_1_/c
a/Å	26.6340(18)
b/Å	10.0262(6)
c/Å	16.7109(10)
α/°	90
β/°	104.840(7)
γ/°	90
Volume/Å^3^	4313.6(5)
Z	4
ρ_calc_(g/cm^3^)	1.468
μ/mm^−1^	3.352
F(000)	1936.0
Crystal size/mm^3^	0.4 × 0.3 × 0.05
Radiation	MoKα (λ = 0.71075)
2Θ range for data collection/°	6.082 to 50
Index ranges	−31 ≤ h ≤ 31, −11 ≤ k ≤ 11, −19 ≤ l ≤ 17
Reflections collected	28,151
Independent reflections	6660 [R_int_ = 0.1162, R_sigma_ = 0.0950]
Data/restraints/parameters	6660/387/560
Goodness-of-fit on F^2^	1.030
Final R indexes [I ≥ 2σ (I)]	R_1_ = 0.0757, wR_2_ = 0.2075
Final R indexes [all data]	R_1_ = 0.1128, wR_2_ = 0.2408
Largest diff. peak/hole/e Å^−3^	0.87/−1.46

**Table 2:** Selected bond lengths (Å) and angles (^o^) for **6**.

Bond Lengths, (Å)		Bond Angles, (^o^)	
Pt1-S1 Pt1-N1 Pt1-O1 Pt1-C11 N2-C12 N2-C13 O1-C12 S1-C13	2.243(5) 2.013(9) 2.045(8) 1.974(12) 1.404(18) 1.371(16) 1.262(15) 1.732(13)	S1-Pt1-O1 S1-Pt1-C11 O1-Pt1-N1 N1-Pt1-C11	93.2(3) 96.4(4) 89.7(4) 80.7(5)

**Table 3:** Transition temperatures (in °C) and enthalpies (kJ·mol^−1^) for platinum(II) complexes ^a,b^.

Complex	Transitions (T/°C, ∆H/kJ∙mol^−1^)
**5**	Cr_1_ 57 (2.5) Cr_2_ 73 (15.1) Cr_3_ 164 (24.5) ^c^ Iso Iso 121 (−11.3) Cr_3_ 89 (−0.2) Cr_2_ 62 (−8.5) Cr_1_
**6**	Cr_1_ 106 (26.0) Cr_2_ 151 (20.4) Iso Iso 139 (−3.9) Cr_3_ 84 (−0.5) Cr_2_ 56 (−2.6) Cr_1_

^a^ Data corresponding to the first heating/cooling cycle. ^b^ Iso—isotropic phase; Cr_1_, Cr_2_, Cr_3_—crystalline phase. ^c^ partial decomposition of the samples (in the 2nd and 3rd heating/cooling cycles, the isotropization temperatures shifted to lower values).

**Table 4:** Absorption (*λ*_abs_^max^) and emission (*λ*_em_^max^) maxima (nm) and luminescence quantum yields (Φ) for platinum complexes in CH_2_Cl_2_, solid and ground states, and PMMA films at room temperature.

Compound	Media	UV-VIS (Solution, 10^−4^ M, CH_2_Cl_2_) λ_abs_^max^ (ε × 10^−3^/M^−1^cm^−1^)	Emission λ_em_^max^ (nm)	Φ (%)	τ (μs)
**5**	CH_2_Cl_2_	285 (26.8), 310 (19.0), 378 (13.4)	480, 519, 557	0.02	<1
	Solid	/	482, 518, 548	13	4
	PMMA (1%)	/	480, 514, 550	28.3	5
	Ground	/	625	7	2
**6**	CH_2_Cl_2_	277 (13.0), 315 (13.4), 369(sh) (6.9)	467, 535, 572, 627(sh)	0.17	<1
	Solid	/	468, 502, 530, 570	2	5
	PMMA (1%)	/	465, 500, 532, 570	5	6
	Ground	/	647	1	3

**Table 5:** Experimental and calculated low-energy absorption maxima (nm), transition energy (eV), oscillator strength (f_calcd_), and major contribution for Pt complexes. H stands for HOMO, and L stands for LUMO.

Complex	λ_exp_/nm	λ_calcd_/nm	E/eV *f*_calcd_	Major Contribution	Assignment
**5**	378	366	3.39 0.2874	H → L (69%) H → L + 1 (10%)	^1^MLCT, ^1^ILCT
**6**	369	369	3.36 0.4704	H → L (91%)	^1^MLCT, ^1^ILCT

**Table 6:** Molecular fragment contribution (%) to selected molecular orbitals of studied complexes in the S_0_ state in CH_2_Cl_2_.

Fragment	H-4	H-3	H-2	H-1	H	L	L + 1
**5**							
1 (Pt)	8.12	88.11	16.06	33.83	15.48	2.43	6.38
2 (Ph)	17.71	2.99	1.69	47.15	0.95	0.59	27.01
3 (PhN)	10.18	1.03	3.01	7.83	2.43	1.86	63.72
4 (PhSN)	10.07	5.69	30.68	3.37	69.44	36.93	1.09
5 (PhNO)	38.11	2.17	36.10	7.10	1.78	55.72	1.74
6 (OC_10_H_21_)	2.93	0.00	4.56	0.00	9.92	0.42	0.00
7 (OC_10_H_21_)	12.88	0.00	7.91	0.72	0.00	2.04	0.06
**6**							
1 (Pt)	86.94	5.01	24.13	25.57	11.70	2.14	6.85
2 (PhFF)	2.69	34.18	12.91	31.26	0.79	0.53	26.58
3 (PhN)	0.91	15.67	4.16	8.47	1.85	1.43	64.19
4 (PhSN)	6.37	6.91	28.35	2.23	72.39	38.63	0.97
5 (PhNO)	3.05	27.02	24.16	25.16	2.01	54.76	1.36
6 (OC_10_H_21_)	0.04	2.12	2.51	0.56	11.24	0.50	0.00
7 (OC_10_H_21_)	0.00	9.08	3.78	6.74	0.02	2.00	0.04

**Table 7:** Calculated emission wavelengths and Pt spin densities in CH_2_Cl_2_.

Complex	λ_em_ (nm)	Pt Spin Density
**5**	591.81	0.206238
**6**	569.33	0.172162

**Table 8:** Lowest vertical singlet and triplet energy excitations as obtained by TDDFT calculations at the S_0_ optimized geometry in CH_2_Cl_2_. ΔE stands for excitation energy, and f stands for oscillator strength.

Complex	λ_exp_ (nm)	λ_calc_ (nm)	f	Main Contribution	Assignment	ΔE (eV)	f	Main Contribution	Assignment
	S_0_ → S_1_					S_0_ → T_n_			
**5**	378	366	0.2874	H → L (69%) H → L + 1 (10%)	^1^MLCT, ^1^ILCT	2.7294	0.00	H-1 → L + 1 (43%) H-4 → L + 1 (16%)	^3^MLCT, ^3^ILCT
						2.8904	0.00	H → L (83%)	^3^ILCT, ^3^MLCT
						3.0382	0.00	H-2 → L (58%) H-4 → L (12%)	^3^ILCT, ^3^MLCT
**6**	369	369	0.4704	H → L (91%)	^1^MLCT, ^1^ILCT	2.8022	0.00	H → L (76%)	^3^MLCT, ^3^ILCT
						2.8193	0.00	H-1 → L + 1 (34%) H-3 → L + 1 (24%) H → L + 1 (10%) H → L (10%)	^3^MLCT, ^3^ILCT
						3.0219	0.00	H-1 → L (37%) H-2 → L (32%)	^3^MLCT, ^3^ILCT

## Data Availability

Data are contained within the article. Further inquiries can be directed to the corresponding author.

## Supplementary Materials

The following supporting information can be downloaded at: https://www.mdpi.com/article/10.3390/molecules30112415/s1, Figure S1. ^1^H-NMR and ^13^C-NMR spectra for 5; Figure S2. IR spectrum for 5; Figure S3. Molecular structure of **6** showing the distortion of decyl chain of the aniline unit disordered into two parts; Figure S4. Crystal packing for compound **6** along *b*-axis; Figure S5. Energy levels of some molecular orbitals of **6** in the S_0_ state in CH_2_Cl_2_; Figure S6. Experimental absorption spectra vs. computed oscillator strengths of studied cyclometalated Pt (II) complexes in CH_2_Cl_2_.
