# Supplementary material for: Investigation of Mechanochromic and Solvatochromic Luminescence of Cyclometalated Heteroleptic Platinum(II) Complexes with Benzoylthiourea Derivatives
Source: Molecules. 2025 May 31;30(11):2415. doi: 10.3390/molecules30112415 (PMC12156917; doi:10.3390/molecules30112415)
Supplement: Supplementary file 1 [file molecules-30-02415-s001.zip › molecules-3684936-supplementary.pdf]

Investigation of mechanochromic and solvatochromic luminescence of cyclometalated heteroleptic platinum(II) complexes with benzoylthiourea derivatives

Monica Iliş[1], Marilena Ferbinţeanu[1], Cristina Tăbleţ[2], Viorel Cîrcu[*,1]

[1]*Department of Inorganic and Organic Chemistry, Biochemistry and Catalysis, University of Bucharest, 4-12 Regina Elisabeta Bld., sector 5, Bucharest 030018, Romania*
[2]*Titu Maiorescu University, Faculty of Pharmacy, Gh. Sincai Bd. 16, 040317 Bucharest, Romania*

Supplementary Information

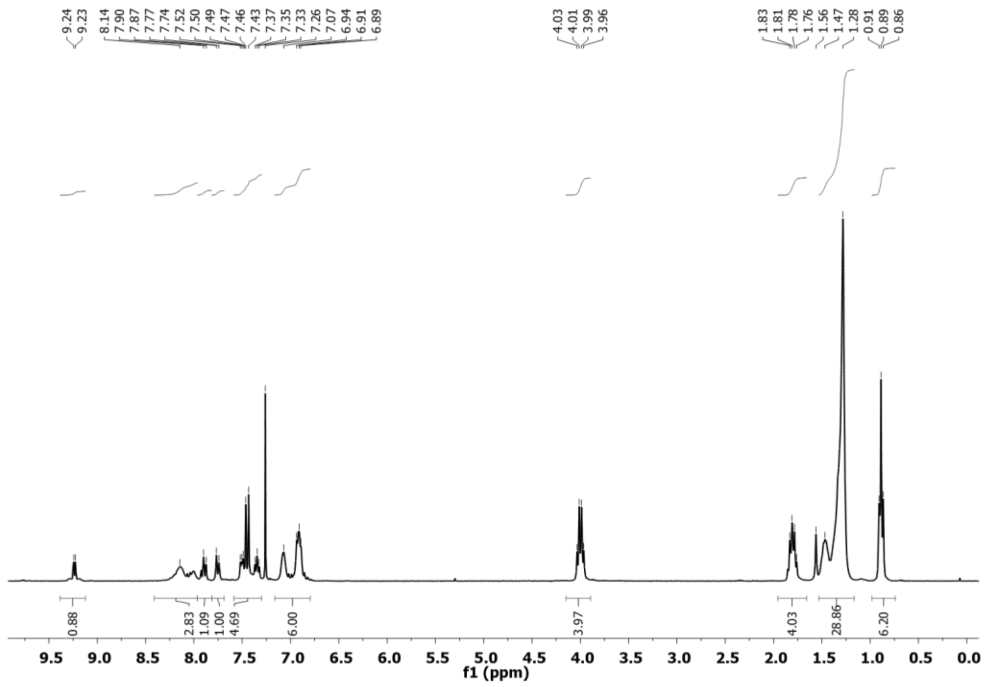

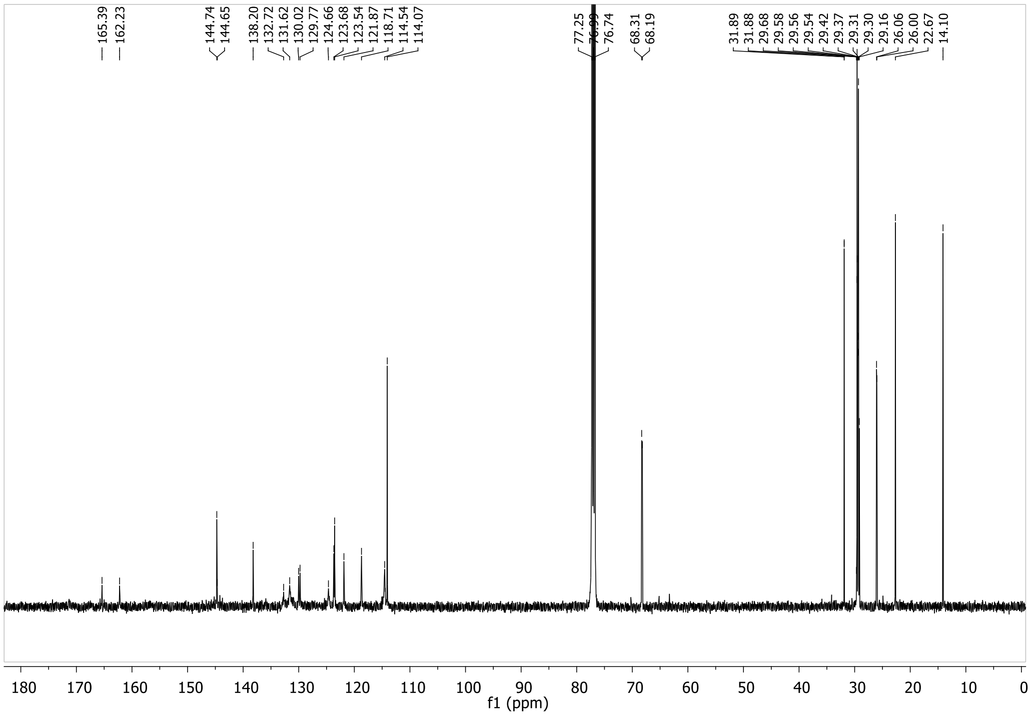

Figure S1. ^{1}H-NMR and ^{13}C-NMR spectra for **5**.

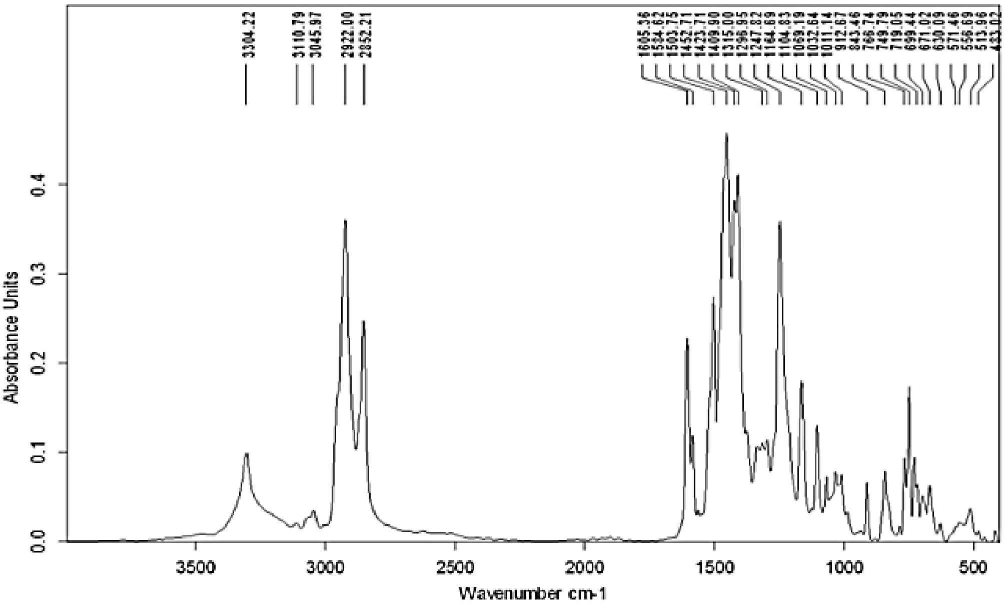

Figure S2. IR spectrum for **5**.

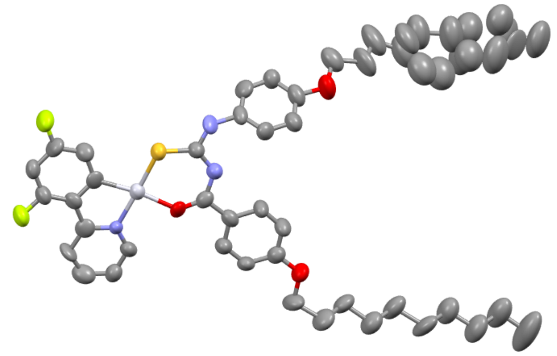

Figure S3. Molecular structure of **6** showing the distortion of decyl chain of the aniline unit disordered into two parts.

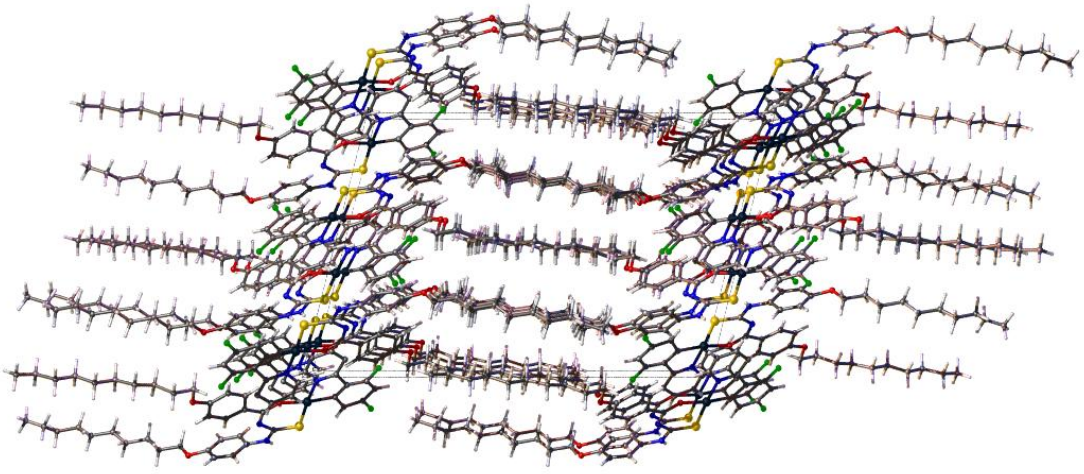

Figure S4. Crystal packing for compound **6** along *b* axis.

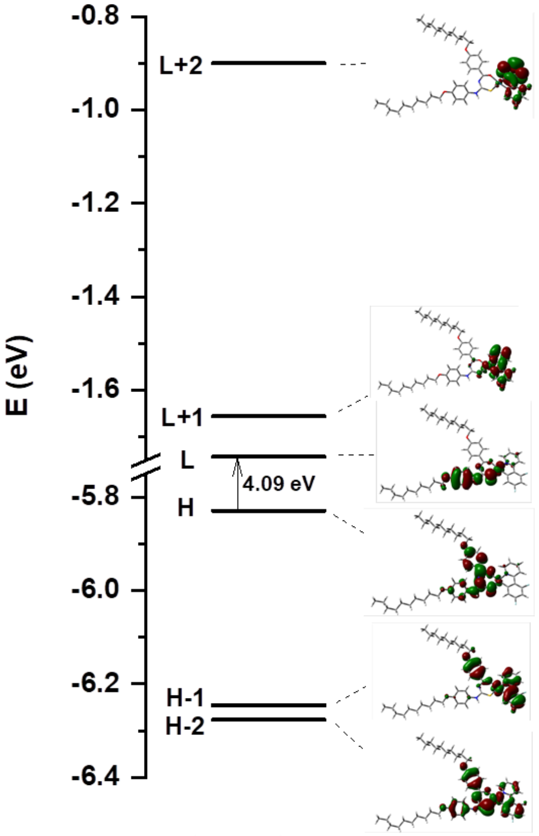

Figure S5. Energy levels of some molecular orbitals of **6** in the S_0 state in CH_2Cl_2.

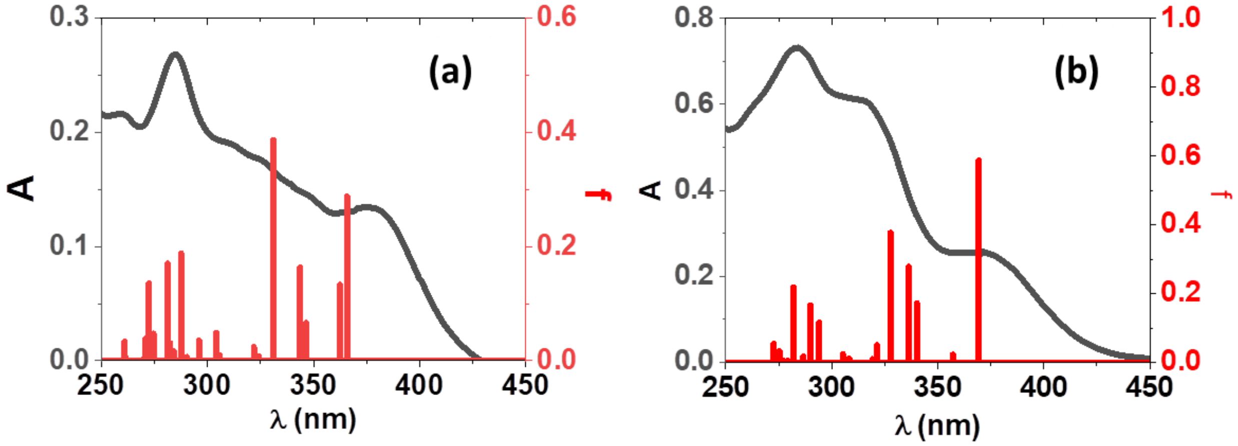

Figure S6. Experimental absorption spectra vs. computed oscillator strengths of studied cyclometalated Pt (II) complexes in CH_2Cl_2.
